# Various Cataract Model Eyes for Wet Lab Training Using the Microwave Surgical Device

**DOI:** 10.7759/cureus.81965

**Published:** 2025-04-09

**Authors:** Kosei Tomita, Masayuki Akimoto, Yoshiaki Ieki, Atsushi Miki

**Affiliations:** 1 Ophthalmology, Kawasaki Medical School, Okayama, JPN; 2 Ophthalmology, Osaka Red Cross Hospital, Osaka, JPN

**Keywords:** microwave surgical device, ophthalmology, phaco-chop, phacoemulsification cataract surgery, wet lab training

## Abstract

Purpose

This study aims to develop a highly reproducible and efficient method for inducing lens nucleus hardening in porcine eyes using a microwave surgical device. The resulting cataract model eyes simulate key aspects of cataract surgery, offering an effective platform for enhancing surgical training.

Methods

Freshly enucleated porcine eyes were prepared. A microwave surgical device with a pencil-type electrode (PE) and needle-type electrode (NE) was utilized. The PE was inserted through a scleral incision 4 mm from the corneal limbus, positioned at the center of the lens nucleus, and activated at 50 mW for 30 seconds to create a nucleus cataract model eye. The NE was inserted through a scleral incision 8 mm from the corneal limbus, directed towards the vitreous cavity, and activated at 10 mW for less than 10 seconds to create a posterior polar cataract model eye. The created nucleus cataract model eyes (n=23) were evaluated using a phacoemulsification machine's cumulative dissipated energy (CDE) value.

Results

Thermal coagulation effectively induced nuclear sclerosis, creating a cloudy nucleus cataract model eye without compromising corneal opacity. The anterior capsule was stained with trypan blue to simulate poor visibility in white cataracts, facilitating capsulorhexis training. The hardened lens nucleus allowed for the practice of phaco-chop and divide-and-conquer techniques with realistic tactile feedback, similar to actual cataract surgery. The mean CDE value of the 23 nucleus cataract model eyes was 8.25, indicating increased nuclear hardness. Additionally, posterior capsule thermal denaturation via the vitreous cavity successfully created a posterior polar cataract model eye. Hydrodelineation and irrigation/aspiration were performed without causing posterior capsular rupture.

Conclusion

This study presents a novel method to create realistic nucleus and posterior polar cataract models using a microwave surgical device in porcine eyes. These models provide a reproducible and effective platform for surgical training, simulating key aspects of cataract surgery, including capsulorhexis under poor visibility and phacoemulsification techniques.

## Introduction

A total of 36 million people are blind, and over 12 million cases are due to cataracts globally [1]. Surgical treatment is the first-line option for cataracts, and it is essential to train surgeons effectively using standardized, high-quality methods to manage this overwhelming patient population. Various training techniques, including wet laboratory (wet lab), dry laboratory, and simulators, have been developed for learning cataract surgery [2-5]. However, each method has limitations that make them different in applicability to actual cataract surgery, and there remains a need for highly reproducible models that closely simulate actual procedures.

There are previously published papers that describe a method of utilizing thermal coagulation created by formalin solution and microwaves and increasing the nuclear hardness of the lens [6,7]. Microwave thermal coagulation can harden and opacify porcine eye lenses. However, since it caused thermal coagulation in substances other than the lens, time and labor had to be spent to prevent corneal opacity by heat, which had become a problem. While microwave heating is effective for thermal coagulation of the lens nucleus, the process must be precisely localized to avoid damaging the cornea.

Because cataracts are most complex at the center, thermal coagulation should induce concentric hardening beginning from the nucleus core outward. Additionally, high-power equipment is necessary to achieve this process efficiently within a short time. In this study, we aim to develop and report an efficient method to thermally induce lens nucleus hardening in porcine eyes using a microwave surgical device, creating cataract model eyes suitable for surgical training.

## Materials and methods

**Figure 1:**
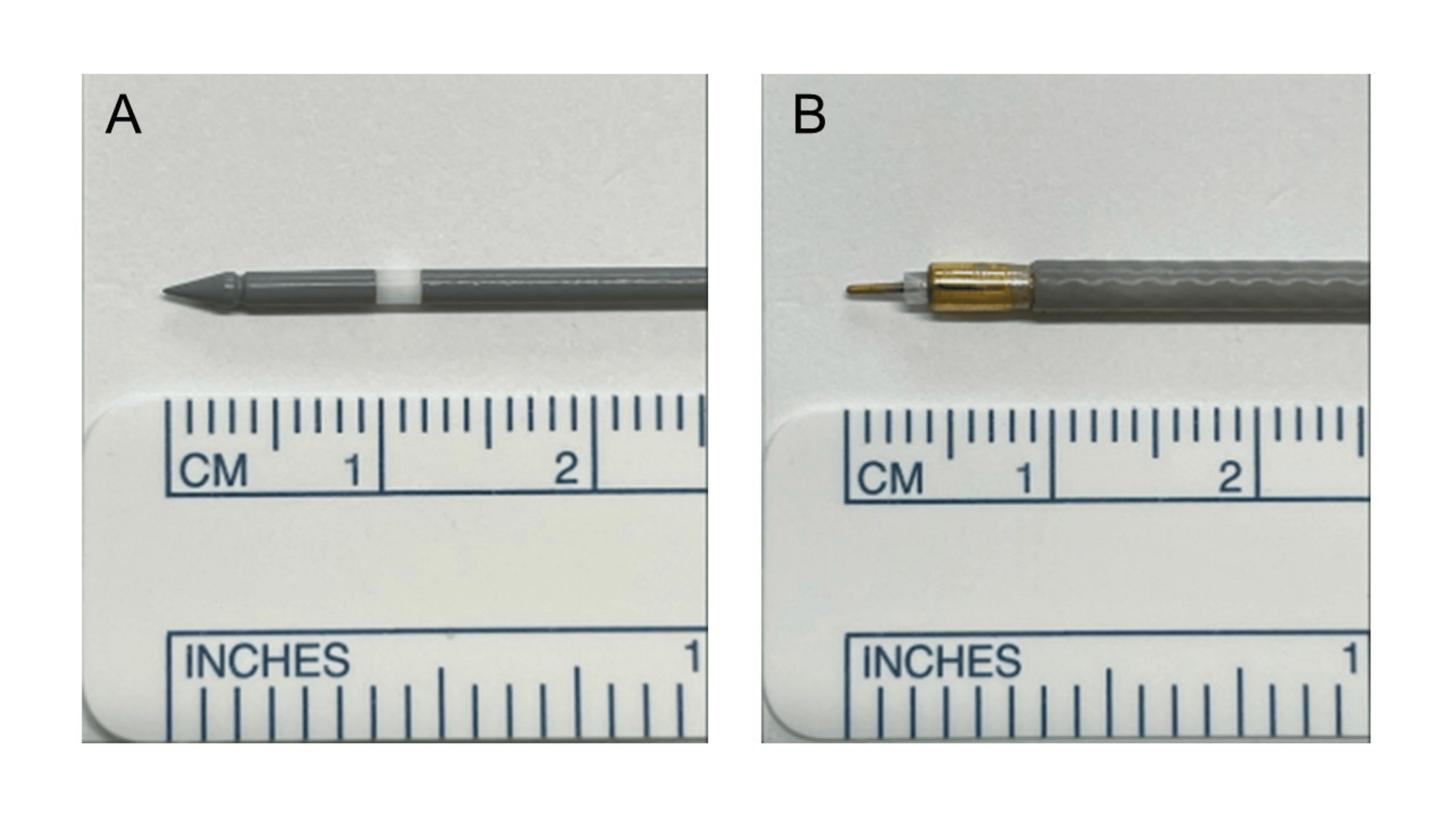
Electrodes of microwave surgical device (A) Pencil-type electrode. (B) Needle-type electrode.

Legally processed porcine eyes obtained from slaughterhouses were used in this study. The prepared porcine eyes were enucleated on the same day as the experiment. The porcine eyes were stored in a container filled with ice-cold water immediately after enucleation to preserve tissue condition. This experimental method does not violate ethical considerations regarding animal experimentation [8]. We used a microwave surgical device (Microtaze; Alfresa Pharma Corporation, Osaka, Japan), and pencil-type electrodes (PE) and needle-type electrodes (NE) were prepared (Figure 1).

The shaft diameter of PE is 1.6 mm, and NE is 2.4 mm. The white band of PE and NE is an insulator. Microwaves are irradiated before and after this white band to cause molecular vibration, which generates dielectric heat. The cumulative dissipated energy (CDE) value was calculated during phacoemulsification using the phaco-chop technique in 23 nucleus cataract model eyes, utilizing a phacoemulsification system (Centurion; Alcon, USA). This study is a single-arm experimental investigation exclusively utilizing porcine eyes obtained from animals processed for meat production. No direct comparative analysis with human ocular tissues was conducted.

Surgical procedure

Nucleus Cataract Model Eye: A 2.4 mm slit knife is used to perforate the sclera in the direction of the lens nucleus, positioned 4 mm away from the corneal limbus of the porcine eye. Next, PE is inserted through the wound, and an insulator of PE is held at the center of the lens nucleus to activate the device. The microwave power is 50 mW, and the activation time is 30 seconds. Posterior Polar Cataract Model Eye: A 2.4 mm slit knife is used to perforate the sclera, positioned 8 mm away from the corneal limbus of the porcine eye, directing the incision towards the vitreous cavity. NE is inserted through the wound and placed in contact with the posterior lens capsule to activate the device. The microwave power is 10 mW, and the operation time is less than 10 seconds.

## Results

Thermal coagulation of the lens nucleus from the center using PE could create a cloudy nucleus cataract model eye (Video 1).

**Video 1:** Creation of nucleus cataract model eye

**Figure 2:**
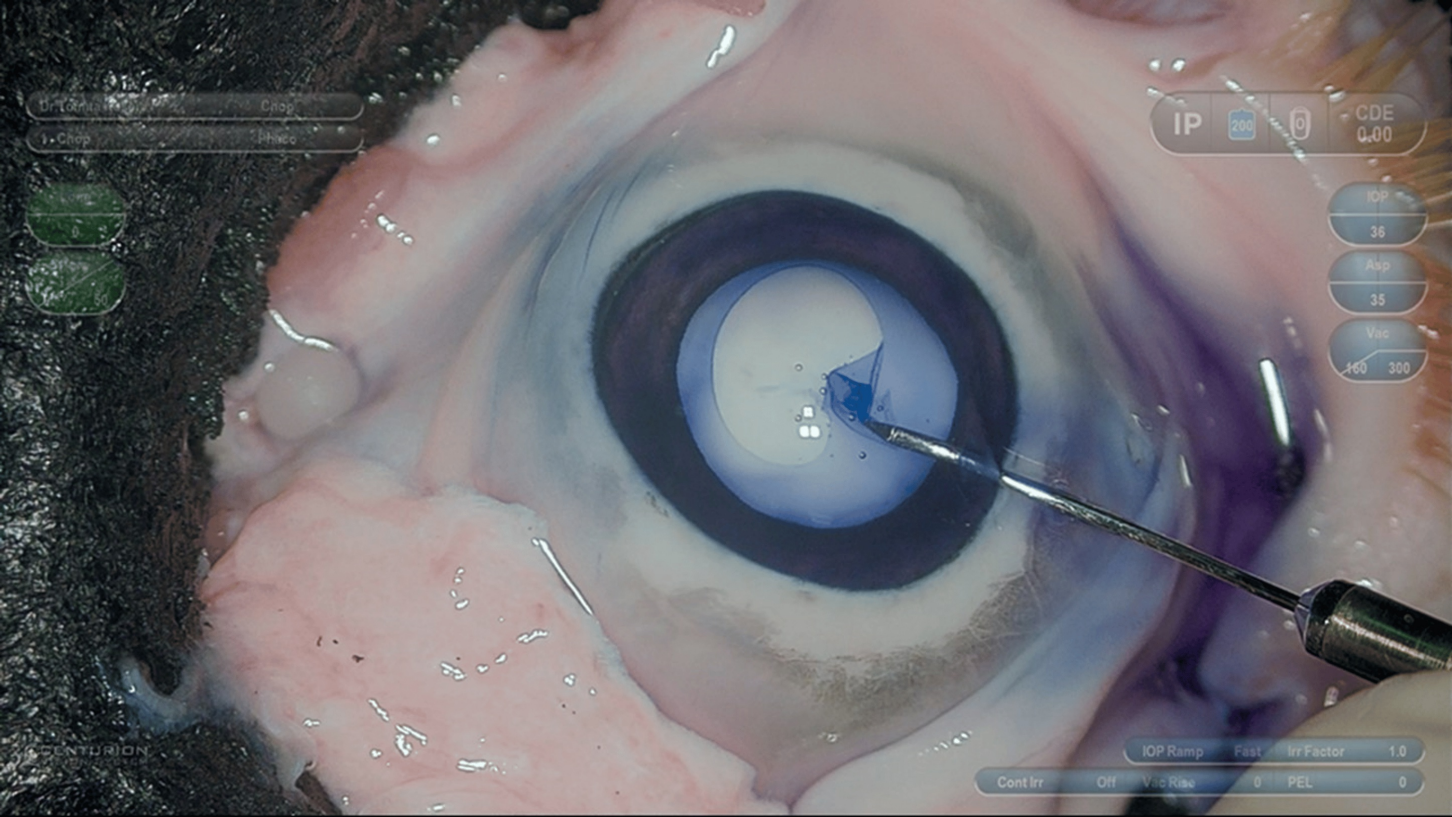
Capsulorhexis with trypan blue We could practice capsulorhexis with trypan blue in poor transparency conditions.

The cornea was not thermally degenerated by creating a nucleus cataract model eye, and corneal opacity did not occur. Anterior capsule staining with trypan blue was performed, and we could practice capsulorhexis in poor transparency conditions, such as white cataracts (Figure 2).

Using a hardened lens nucleus, we could practice phaco-chop (Video 2) and divide-and-conquer techniques (Video 3).

**Video 2:** Phaco-Chop technique

**Video 3:** Devide-and-conquer technique

The nucleus cataract model eye maintains a higher nuclear hardness, so we can learn to maneuver the foot pedal work with the same handling feel as actual cataract surgery (Video 4).

**Video 4:** Foot pedal work

**Figure 3:**
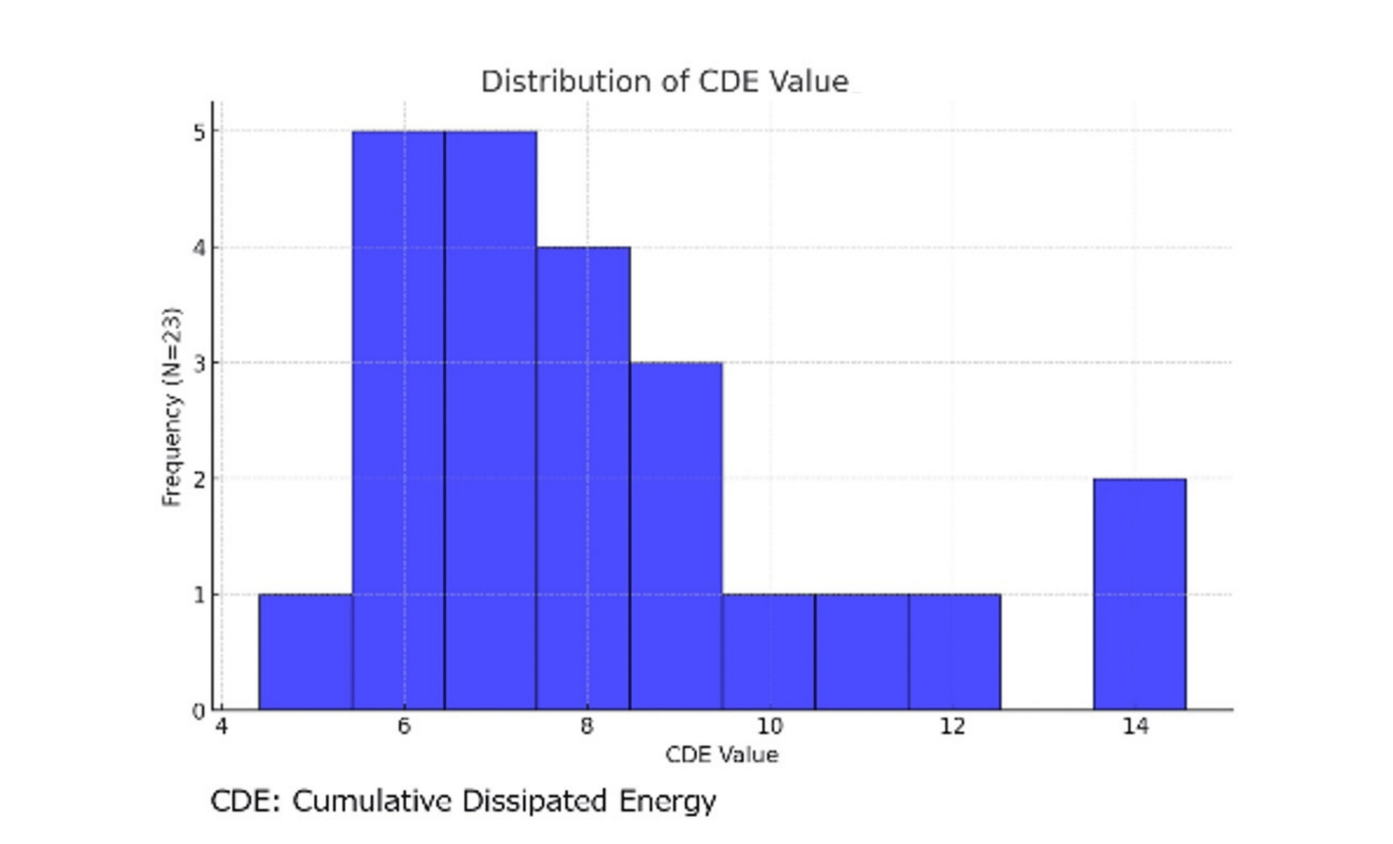
Cumulative dissipated energy of nucleus cataract model eye under phaco-chop technique The cumulative dissipated energy (CDE) value was calculated for 23 nucleus cataract model eyes under the phaco-chop technique. The CDE value was averaged at 8.25.

The 23 nucleus cataract model eyes were created and evaluated for CDE values under the phaco-chop technique. The mean CDE was 8.25 (Figure 3).

Of the 23 nucleus cataract model eyes, posterior capsular rupture (PCR) occurred in nine cases. No cases of zonular dialysis or lens subluxation were observed during phacoemulsification. The lens nucleus was removed entirely in cases without PCR. Thermal denaturing only the posterior capsule of the lens via the vitreous cavity using NE, we could create a posterior polar cataract model eye (Video 5).

**Video 5:** Creation of posterior polar cataract model eye

The posterior polar cataract model eye was performed by hydro delineation, and the opacity was removed by irrigation and aspiration. After this procedure, the posterior capsule remained intact without leading to posterior capsular rupture (Video 6).

**Video 6:** No posterior capsular rupture in posterior polar cataract model eye

## Discussion

Porcine and sheep eyes are suitable sizes for wet labs in ophthalmic training [2,9]. However, they are too young to mimic senile cataracts in nature. Therefore, they are inadequate for learning nucleofractis techniques and phacoemulsification, which are important processes in cataract surgery. In this study, we attempted to create a nucleus cataract model eye and a posterior polar cataract model eye using a microwave surgical device not made for the ophthalmic field. This device uses microwaves to provide high-power, localized hemostasis, and coagulation, and it is actively used in treating hepatic tumors as microwave coagulation therapy [10,11]. Since nucleus cataract model eyes had the mean CDE value of 8.25 in phacoemulsification, this procedure could form the nuclear cataract model eye with a certain hardness level without corneal opacity.

Posterior polar cataract is an uncommon condition that is at risk of posterior capsular rupture during hydrodissection [12]. By performing hydrodelineation in this posterior polar cataract model eye, separating the outer shell and the endonuclease, and processing each component separately, we were able to remove the lens without causing posterior capsular rupture. Since posterior polar cataract occurs less frequently than typical nuclear cataracts, it is encountered less often in clinical practice. Therefore, utilizing the posterior polar cataract model eye for surgical training can be beneficial in preparing for such cases in actual clinical settings.

Training in cataract surgery using a surgical simulator has been shown to reduce complication rates and improve trainees' technical skills, thereby enhancing ophthalmic surgical education [5,13]. However, there is a concern that disparities in surgical education may arise between institutions that can afford expensive simulators and those that cannot. Therefore, there remains a high demand for methods to further enhance wet laboratory training using porcine eyes, which are more cost-effective than simulators, to better replicate the actual cataract surgery environment. In this study, we could not perform staining, imaging, or histological analysis of opacity in either the nuclear cataract model eye or the posterior polar cataract model eye. Furthermore, direct comparisons between human and porcine eyes were not conducted, including potential differences in lens thickness, nucleus consistency, and capsular anatomy. These factors may influence the applicability of each model in wet lab training.

**Figure 4:**
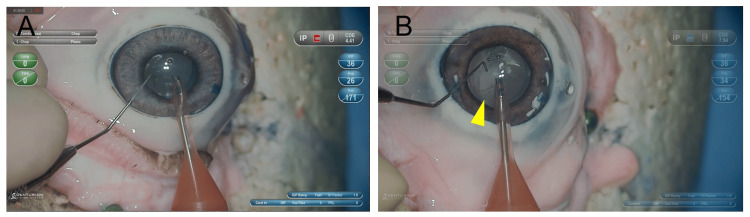
Occurring posterior capsular rupture or not (A) No posterior capsular rupture after phacoemulsification. (B) Posterior capsular rupture was observed due to water pressure (arrowhead).

A limitation associated with this procedure is puncturing the lens capsule's equatorial part when creating a nucleus cataract model eye. Because of this problem, the posterior capsule had been severely damaged by water pressure in nine of 23 cases (Figure 4).

We punctured the lens capsule at the equator to insert PE. This procedure might transmit stress to the posterior capsule due to the lens capsule's anatomical continuity. The posterior capsule rupture was presumed to be caused by water pressure. However, whether the rupture resulted from the pressure generated during phacoemulsification or hydrodissection remains unclear. Hydrodissection was successfully performed on nucleus cataract model eyes. However, it was difficult to visually confirm whether the hydrodissection was fully completed due to the high lens opacity. To reduce the incidence of posterior capsule rupture, a 2.4 mm slit knife was used to perforate the sclera in the direction of the lens nucleus at a point 2 mm away from the corneal limbus, and PE was activated. However, the heat generated during activation adversely affected the cornea, resulting in corneal opacity. One possible application may be to switch to extracapsular cataract extraction by anticipating PCR. Wet labs using microwave surgical devices also make it possible to practice recovery approaches in the event of complications, which cannot be done with conventional laboratories. However, this study did not include a direct comparison or statistical analysis with conventional training methods such as unprocessed porcine eyes or traditional wet lab training. Considering the need to prepare a microwave surgical device, issues regarding its accessibility and distribution remain.

This procedure may create a situation more similar to the environment in cataract surgery than in conventional laboratories for ophthalmic training. However, this microwave surgical device was not specifically designed for ophthalmic use. It was originally developed for applications in internal medicine and general surgery, making it not reasonable for use exclusively in an ophthalmic wet lab. Additionally, the procedure requires rupturing the lens capsule at the equator to puncture the lens nucleus, preventing the creation of a fully intact nucleus cataract model eye. Going forward, we would like to develop a microwave surgical device specifically designed for ophthalmic wet labs, optimizing the electrode shape to create a nucleus cataract model eye without rupturing the lens capsule.

## Conclusions

This study introduces a novel method for generating realistic nucleus and posterior polar cataract models in porcine eyes using a microwave surgical device. These models provide a reproducible and effective training platform for cataract surgery, such as performing capsulorhexis under limited visibility and phacoemulsification techniques. These findings may suggest that thermally induced cataract models significantly enhance cataract surgery training in ophthalmic education.
